# Development of a loop-mediated isothermal amplification coupled lateral flow dipstick targeting *erm*(41) for detection of *Mycobacterium abscessus* and *Mycobacterium massiliense*

**DOI:** 10.1186/s13568-019-0734-4

**Published:** 2019-01-23

**Authors:** Dongxin Liu, Wencong He, Mingxia Jiang, Bing Zhao, Xichao Ou, Chunfa Liu, Hui Xia, Yang Zhou, Shengfen Wang, Yuanyuan Song, Yang Zheng, Qian Chen, Jiale Fan, Guangxue He, Yanlin Zhao

**Affiliations:** 1 National Institute for Communicable Disease Control and Prevention, Chinese Center for Disease Control and Prevention, Changping, Changbai Road 155, Beijing, 102206 China; 2 Institute for Communicable Disease Control and Prevention, Qinghai Provincial Center for Disease Control and Prevention, Chengdong District, Bayi Road 55, Xining, 810007 China; 3 National Tuberculosis Reference Laboratory, Chinese Center for Disease Control and Prevention, Changping, Changbai Road 155, Beijing, 102206 China; 4 Science and Technology Department, Chinese Center for Disease Control and Prevention, Changping, Changbai Road 155, Beijing, 102206 China

**Keywords:** *Mycobacterium abscessus*, *Mycobacterium massiliense*, Drug susceptibility testing, LAMP, *erm*(41) gene, Diagnosis

## Abstract

*Mycobacterium abscessus* (*M. abscessus*) and *Mycobacterium massiliense* (*M. massiliense*) are major pathogens that cause post-surgical wound infection and chronic pulmonary disease. Although they are closely related subspecies of *M. abscessus* complex, their infections are associated with different drug-resistance and cure rate. In the present study, a loop-mediated isothermal amplification (LAMP) coupled with lateral flow dipstick (LFD) method was developed to simultaneous detect *M. abscessus* and *M. massiliense*, via specific *erm*(41) gene. The amplification was carried out at 65 °C for only 60 min, and the results could be visualized on a lateral flow strip. Positive results only occurred in *M. abscessus* and *M. massiliense*, no cross-reaction with other mycobacterial species was observed. Therefore, the cost-effective MABC (*M. abscessus* complex)–LAMP–LFD method developed here was able to correct the diagnose of *M. abscessus* and *M. massiliense* infection in a short time. Thus, this method could be used to guide clinicians in treatment of *M. abscessus* group infections.

**Electronic supplementary material:**

The online version of this article (10.1186/s13568-019-0734-4) contains supplementary material, which is available to authorized users.

## Introduction

Rapidly growing mycobacteria (RGM) are ubiquitous environmental microorganisms (Brown-Elliott and Wallace 2002) and the prevalence of pulmonary infection due to RGM is increasing worldwide. Within the RGM, the *Mycobacterium abscessus* complex is a prominent cause of lung disease in patients with chronic pulmonary disease and cystic fibrosis (Chan et al. 2010; Zelazny et al. 2009). Based on divergence of *rpoB* sequences, *M. abscessus* complex is thought to be comprised of three subspecies—*Mycobacterium abscessus*, *Mycobacterium massiliense* and *Mycobacterium bolletii* (Adekambi et al. 2006; Howard 2013). Of the three, *M. bolletii* is rarely isolated, while *M. massiliense* and *M. abscessus* are major pathogens (Benwill and Wallace 2014; Brown-Elliott et al. 2015).

Infections caused by *M. abscessus* complex are often difficult to treat, because these mycobacteria are intrinsically resistant not only to the traditional anti-tuberculous drugs but also to most currently available antimicrobial agents (Kim et al. 2015). Macrolides, such as clarithromycin and azithromycin, are considered to be the cornerstone of antimicrobial treatment strategies targeting *M. abscessus* complex infections (Nash et al. 2006). However, some *M. abscessus* strains have displayed intrinsic resistance to clarithromycin due to a mutation at position A2058 or A2059 in the *rrl* gene region. Furthermore, poor treatment outcomes of *M. abscessus* infections have also been attributed to inducible resistance conferred by the erythromycin ribosomal methylase gene, *erm*(41) (Nash et al. 2009). In contrast, few *M. massiliense* strains have shown inducible resistance to clarithromycin because of a large (274-bp) fragment deletion in *M. massiliense*’s *erm*(41) gene (Kim et al. 2010). To exploit this genetic difference, PCR-based assays of this conserved deletion in *erm*(41) have been proposed as a simple method to distinguish *M. abscessus* from *M. massiliense*, based on the fragment size of the resulting amplification product (Kim et al. 2010).

Due to the different drug susceptibility and treatment outcomes of *M. abscessus* and *M. massiliense*, identification of *M. abscessus* complex at the species-level in clinical settings is of critical importance, because it can provide a first indication of antibiotic susceptibility and suggest the appropriate drug therapy (Kim et al. 2015). Thus, it is necessary to establish a rapid, accurate and simple method to identify *M. abscessus* and *M. massiliense*. Although targeted gene sequencing (*hsp65*, *rpoB* and *secA1*) and PCR-based assays (*erm*(41)) have been used for identification of *M. abscessus* and *M. massiliense* (Shallom et al. 2013; Zelazny et al. 2009), these two methods are time-consuming in a routine clinical microbiology laboratory and often require instrumentation that may not be available in resource-limited settings.

The loop-mediated isothermal amplification (LAMP) method provides several advantages over targeted gene sequencing or PCR-based methods. Most importantly, it is capable of amplifying DNA rapidly under isothermal conditions at 60–65 °C, it means that an incubator or water bath is sufficient for performing LAMP assays (Hayashida et al. 2015), moreover, the amplification products can also be analyzed using several common methods, such as gel electrophoresis, colorimetric agents, real-time turbidimeters, and a gold nanoparticle-based immunochromatographic technique, thereby reducing the barriers to implementation of this molecular amplification method in resource-limited settings.

In this study, we devised a LAMP assay combined with a lateral flow dipstick (LAMP–LFD) for simultaneous, rapid and visual detection of *M. abscessus* and *M. massiliense* using one target gene in a single test. The performance and limitations of MABC–LAMP–LFD method in detecting *M. abscessus* and *M. massiliense* from clinical isolates was also evaluated. In addition, we conducted drug susceptibility testing (DST) on *M. abscessus* complex clinical isolates to confirm their drug susceptibility patterns.

## Materials and methods

### Reagents and instruments

The Loopamp kits were purchased from Eiken Chemical Co., Ltd. (Japan). Lateral flow dipstick and running buffer were purchased from HaiTaiZhengYuan Technology Co., Ltd. (Beijing, China). Biotin-14-dCTP was obtained from Thermo Scientific. Co., Ltd (Shanghai, China). Clarithromycin, azithromycin, amikacin, levofloxacin, moxifloxacin, and gatifloxacin were obtained from Sigma-Aldrich (USA). Realtime turbidimeter was purchased from LanPu Biotechnology Co., Ltd. (Beijing, China).

### Mycobacterial strains and identification

A total of 134 mycobacterial strains obtained from Guangzhou Chest Hospital and 1 *M. abscessus* reference strain (ATCC 19977) were used in this study. The genomic DNA of mycobacterial strains were extracted using a CTAB-phenol–chloroform extraction method. Then all isolated strains were subjected to *16S rRNA*, *rpoB*, *hsp65* and *ITS* gene sequencing that allow precise discrimination of mycobacterial species. 51 isolates were determined to be *M. abscessus*, while 53 isolates were identified as *M. massiliense* and 30 isolates were from other mycobacterial species (Table 1).

**Table 1:** Strains used in this study

Bacterial species	Source of strains	No. of strains
*M. abscessus*	ATCC 19977	1
	Isolated (Guangzhou Chest Hospital)	51
*M. massiliense*	Isolated (Guangzhou Chest Hospital)	53
*M. tuberculosis*	Isolated (Guangzhou Chest Hospital)	10
*M. fortuitum*	Isolated (Guangzhou Chest Hospital)	5
*M. grodon*	Isolated (Guangzhou Chest Hospital)	5
*M. kansasii*	Isolated (Guangzhou Chest Hospital)	3
*M. intracellulare*	Isolated (Guangzhou Chest Hospital)	5
*M. avium*	Isolated (Guangzhou Chest Hospital)	2
*Staphylococcus aureus*	Isolated (China CDC)	2
*Streptococcus pneumoniae*	Isolated (China CDC)	3
*Corynebacterium diphtheriae*	Isolated (China CDC)	2

### Drug susceptibility testing for *M. abscessus* and *M. massiliense*

Susceptibility testing was carried out using CLSI-recommended broth microdilution MIC method (Clinical and Laboratory Standards Institute 2011). We tested six antimicrobial agents for *M. abscessus* and *M. massiliense*, including clarithromycin, azithromycin, amikacin, levofloxacin, moxifloxacin and gatifloxacin. Clarithromycin MICs results were determined after 3 and 14 days of incubation, while the other antimicrobial agents’ MICs were determined after 3 days incubation. MIC breakpoints for antibacterial agents recommended by CLSI (Clinical Laboratory Standard Institute) were strictly followed.

### PCR assay of *erm*(41) gene

The region of the *erm*(41) was amplified using the primers *erm*F (5′-GAC CGG GGC CTT CTT CGT GAT-3′) and *erm*R (5′-GAC TTC CCC GCA CCG ATT CC-3′) with 52 *M. abscessus* and 53 *M. massiliense* (Kim et al. 2010). The PCR cycling conditions consisted of an initial denaturation at 95 °C for 5 min, 35 cycles of denaturation at 95 °C for 1 min, annealing at 66 °C for 1 min, and extension at 72 °C for 1.5 min, with a final extension at 72 °C for 10 min. PCR amplification products were analyzed by electrophoresis on a 2% agarose gel.

### Primers design for MABC–LAMP–LFD assay

**Fig. 1:**
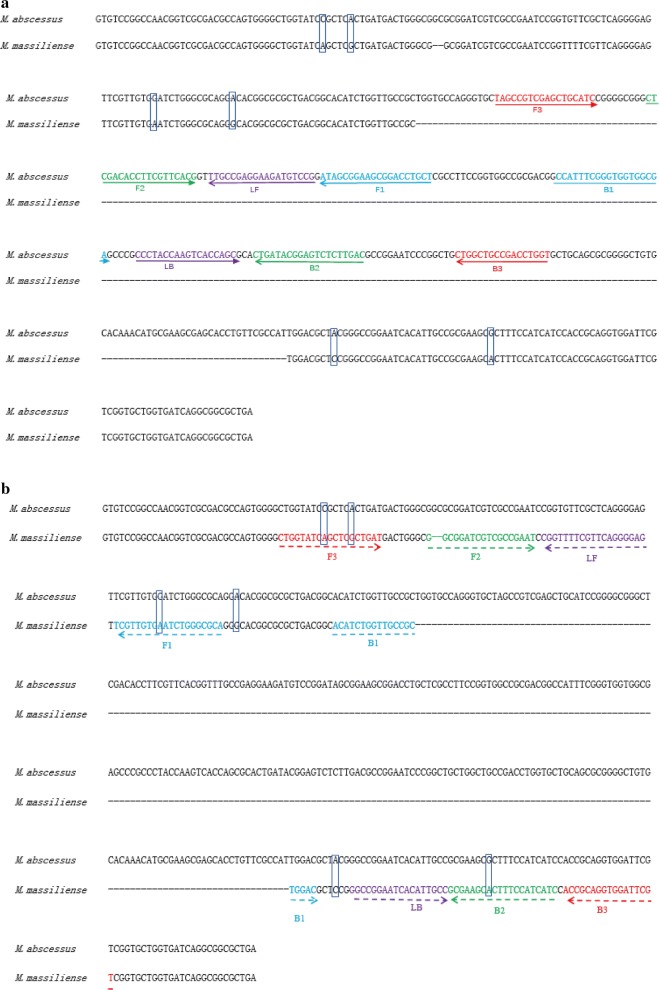
Sequence alignment of *M. abscessus* and *M. massiliense erm*(41) gene and primers location of *M. abscessus* and *M. massiliense*. **a** Primers design for *M. abscessus*, **b** Primers design for *M. massiliense*

**Fig. 2:**
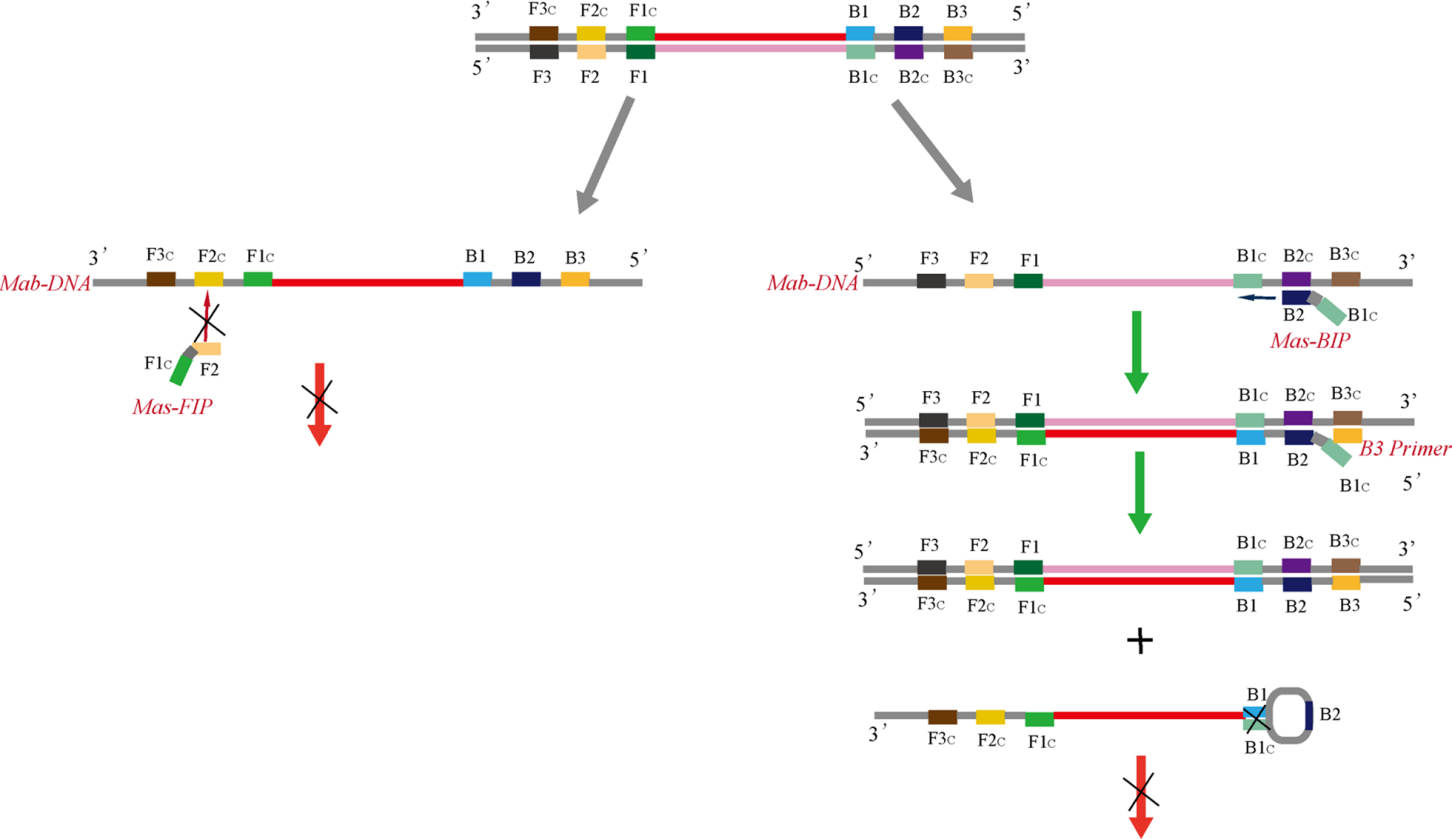
Schematic diagram of *M. massiliense*-LAMP primers inability to amplify the *M. abscessus erm*(41) sequence

The nucleotide sequence of *erm*(41) gene of *M. abscessus* (GenBank accession number: KT185493.1) and *M. massiliense* (GenBank accession number: FJ358487.1) were used as the reference nucleic acid sequences. Sequence alignment revealed the *erm*(41) sequence of *M. abscessus* was 522 bp; however, the *erm*(41) sequence of *M. massiliense* contained only 246 bp due to two deletions (one is 2 bp and the other one is 274 bp). *M. abscessus*-specific LAMP primer pairs were designed targeting the sequence containing the 274 bp deletion in *M. massiliense*. To design *M. massiliense*-specific primers, the *M. massiliense* primer F2 was designed across the 2-bp deletion and the primer B1 across the 274 bp-deletion. Since FIP cannot bind with *M. abscessus erm*(41) gene, and while BIP could bind with *M. abscessus erm*(41) gene, it cannot form a ring, *M. massiliense*-LAMP primers cannot catalyze an amplification reaction in presence of *M. abscessus* DNA template. The details of primer design, primer sequences, and positions are displayed in Table 2 and Fig. 1, while the schematic diagram of *M. massiliense*-LAMP primers inability to amplify the *M. abscessus erm*(41) sequence is displayed in Fig. 2.

**Table 2:** Primers used in this study

Primers name	Sequences and modifications (5′ to 3′)	Length	Detected strains
mab-F3	TAGCCGTCGAGCTGCATC	18	*M. abscessus*
mab-B3	ACCAGGTCGGCAGCCAG	17	
mab-LF	CGGACATCTTCCTCGGCAA	19	
mab-LB*	FAM-CCCTACCAAGTCACCAGC	18	
mab-FIP	AGCAGGTCCGCTTCCGCTATCTCGACACCTTCGTTCACG	39	
mab-BIP	CCATTTCGGGTGGTGGCGAGTCAAGAGACTCCGTATCAG	39	
mas-F3	CTGGTATCAGCTCGCTGAT	19	*M. massiliense*
mas-B3	ACGAATCCACCTGCGGT	17	
mas-LF*	Dig-TCCCCTGAACGAAAACCG	18	
mas-LB	GGCCGGAATCACATTGCC	18	
mas-FIP	TGCGCCCAGATTCACAACGAGGCGGATCGTCGCCGAAT	38	
mas-BIP	ACATCTGGTTGCCGCTGGACGATGATGGAAAGTGCTTCGC	40

### The standard LAMP assay

In order to examine the suitability of *M. abscessus*-LAMP primers and *M. massiliense*-LAMP primers, the single LAMP reaction either for *M. abscessus* strains or *M. massiliense* strains were conducted as the standard LAMP assay. The LAMP reaction mixtures were performed using the Loopamp DNA amplification Kit in a volume of 25 μl containing 0.4 μM each of outer primers F3 and B3, 1.2 μM each of loop primers LF (LF*) and LB (LB*), 2.4 μM each of inner primers FIP and BIP. 12.5 μl 2× reaction mix, 1.25 μl of *Bst* DNA polymerase (10 U) and 1 μl DNA template. Turbidimeters (LA-320C) were used for confirming the amplification of LAMP. Then, the LAMP reaction mixtures were conducted at a fixed temperature ranging from 61 to 67 °C for 60 min to determine the optimal temperature. The DNA templates of *M. massiliense* and *M. tuberculosis* were used as negative control for *M. abscessus*-LAMP reaction, *M. abscessus*- and *M. tuberculosis*-genomic template were used as negative control for *M. massiliense*-LAMP reaction. Mixtures with 1 μl double distilled water were used as blank control.

### The multiplex LAMP assay

To ensure the simultaneous amplification of the two sets of LAMP primers in a single vessel, the concentration of primers was adjusted on the basis of the standard LAMP assay. Briefly, the reaction mixtures of LAMP were performed using the Loopamp DNA amplification Kit in a final volume of 25 μl containing 0.4 μM each of outer primers mab-F3, mab-B3, mas-F3 and mas-B3, 0.4 μM each of loop primers mab-LF, mab-LB*, mas-LF* and mas-LB. 1.2 μM each of inner primers mab-FIP, mab-BIP, mas-FIP and mas-BIP. 12.5 μl 2× reaction mix, 1.25 μl of Bst DNA polymerase (10 U), 0.1 μl of 50 nM biotin-14-dCTP, and 1 μl DNA template. The reactions were conducted at 65 °C for 60 min.

### Detection of *M. abscessus*- and *M. massiliense*-LAMP products in multiplex LAMP assay

As primer mab-LB* labeled with FITC at the 5′ end, primer mas-LF* labeled with digoxin, and the reaction mixture contained bioton-14-dCTP. After amplification, the products of *M. abscessus*-LAMP reaction labeled with FITC and biotin, however, the amplicons of *M. massiliense*-LAMP reaction labeled with digoxin and biotin. To specifically detect *M. abscessus*- and *M. massiliense*-LAMP products, the lateral flow dipstick method was selected. At the lateral flow dipstick, the biotin-labeled LAMP amplicons could form a complex with SA-DNPs via biotin–streptavidin interactions. The biotin/LAMP complexes were captured at the TL I by interaction between anti-FITC and FITC, and at the TL II by interaction between anti-Dig and Dig, whereas the SA-DNPs that did not form complexes were immobilized at the CL by interaction between biotin and streptavidin. As a result, FITC/LAMP/SA-DNPs complexes, Dig/LAMP/SA-DNPs complexes, and non-complexed SA-DNPs were indicated by crimson red lines at the TL I, TL II, and CL, respectively.

### Limit of detection of the MABC–LAMP–LFD assay

The limit of detection (LoD) was determined using *M. abscessus* (ATCC 19977)- and *M. massiliense* (Isolated-*massiliense*-001)-genomic DNA template serial dilutions (10 ng, 1 ng, 100 pg, 10 pg, 1 pg, 100 fg, 10 fg, 1 fg).

### Sensitivity and specificity of the MABC–LAMP–LFD assay

To test the specificity and sensitivity of MABC–LAMP–LFD method, the reactions were conducted under the condition described above with DNA templates of 20 *M. abscessus* strains, 20 *M. massiliense* strains, 15 other mycobacterial strains and 7 non-mycobacterial isolates. In addition, 16 mixed (1:1) DNA templates of *M. massiliense* and *M. abscessus* were tested.

## Results

### Drug susceptibility profile of *M. abscessus* and *M. massiliense*

Drug susceptibility testing was performed on 52 *M. abscessus* strains and 53 *M. massiliense* isolates. Different drug susceptibility profiles of *M. abscessus* and *M. massiliense* were observed (Table 3, Additional file 1). For *M. abscessus*, the clarithromycin MICs showed an obvious increase from day 3 to day 14. In contrast, the susceptibility to clarithromycin in *M. massiliense* showed almost no change, suggesting *M. massiliense* lacks the inducible clarithromycin resistance found in *M. abscessus*.

**Table 3:** Results of drug susceptibility testing against *M. abscessus* and *M. massiliense*

Drugs	Species	No. (%) of strains			χ^2^	*P* value
		Susceptible	Intermediate	Resistant		
Clarithromycin_3d_	*M. abscessus*	49 (94.2)	1 (1.9)	2 (3.8)	1.010	0.60
	*M. massiliense*	51 (96.2)	0 (0.0)	2 (3.8)		
Clarithromycin_14d_	*M. abscessus*	9 (17.3)	0 (0.0)	43 (82.7)	65.847	< 0.001
	*M. massiliense*	51 (96.2)	0 (0.0)	2 (3.8)		
Azithromycin	*M. abscessus*	51 (98.1)	–	1 (1.9)	0.000	1.00
	*M. massiliense*	52 (98.1)	–	1 (1.9)		
Amikacin	*M. abscessus*	52 (100.0)	0 (0.0)	0 (0.0)	16.178	< 0.001
	*M. massiliense*	39 (73.6)	12 (22.6)	2 (3.8)		
Levofloxacin	*M. abscessus*	0 (0.0)	0 (0.0)	52 (100.0)	0.000	1.00
	*M. massiliense*	0 (0.0)	0 (0.0)	53 (100.0)		
Moxifloxacin	*M. abscessus*	0 (0.0)	5 (9.6)	47 (90.4)	0.542	0.72
	*M. massiliense*	0 (0.0)	3 (5.7)	50 (94.3)		
Gatifloxacin	*M. abscessus*	0 (0.0)	7 (13.5)	45 (86.5)	1.770	0.32
	*M. massiliense*	0 (0.0)	3 (5.7)	50 (94.3)		

### Presence of the *erm*(41) gene in clinical isolates

**Fig. 3:**
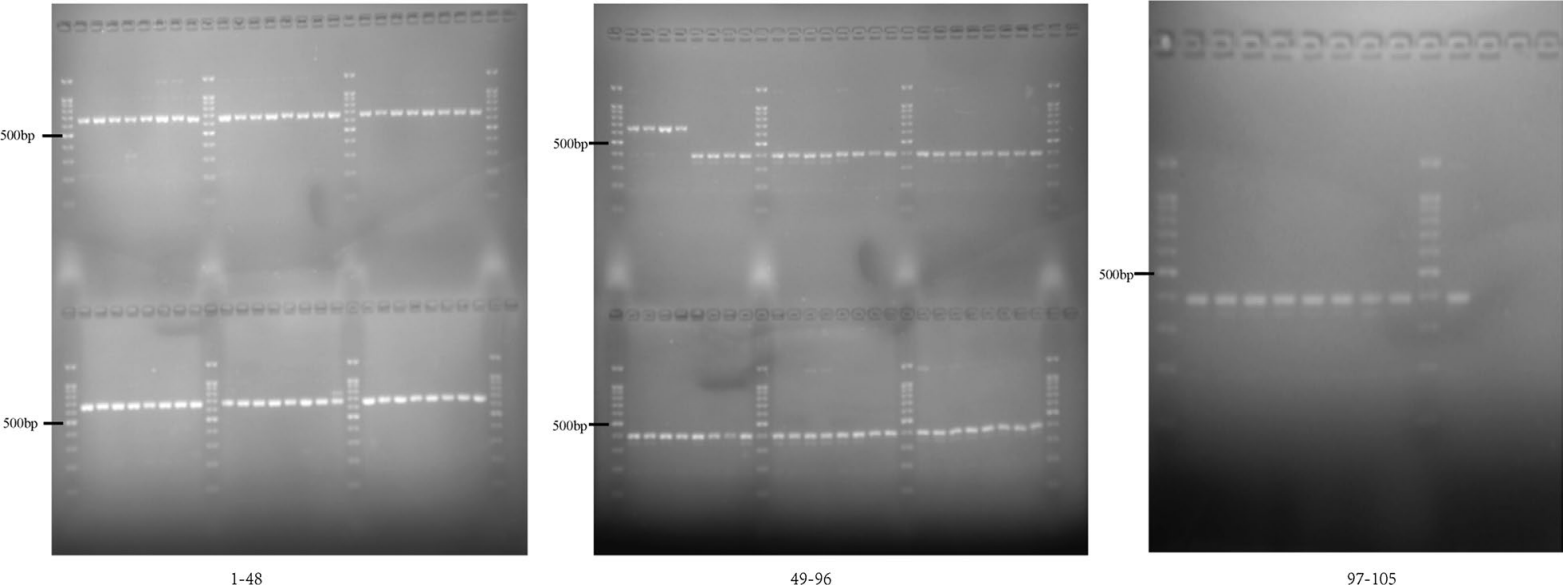
PCR amplified products from *M. abscessus* and *M. massiliense*. Amplified *erm*(41) products of *M. abscessus* were larger than those of *M. massiliense*. 1–52, *M. abscessus*; 53–105, *M. massiliense*

The novel *erm*(41) gene was found to be unique to the *M. abscessus* complex. Full-length *erm*(41) gene in *M. abscessus* has previously been shown to confer inducible macrolide resistance, decreasing the effectiveness of clarithromycin therapy. However, the *erm*(41) gene in *M. massiliense* contains two deletions (274-bp and 2-bp) that result in a nonfunctional *erm*(41) gene, which explains the lack of change between the observed clarithromycin MICs for *M. massiliense* on day 3 and day 14. Two different sized products were found after PCR amplification of DNA templates using the primers of *erm*F and *erm*R. Specifically, the amplification products were about 680 bp from the *M. abscessus* DNA template and 400 bp from the *M. massiliense* DNA template (Fig. 3).

### Optimal amplification temperature and specificity of standard LAMP assay

**Fig. 4:**
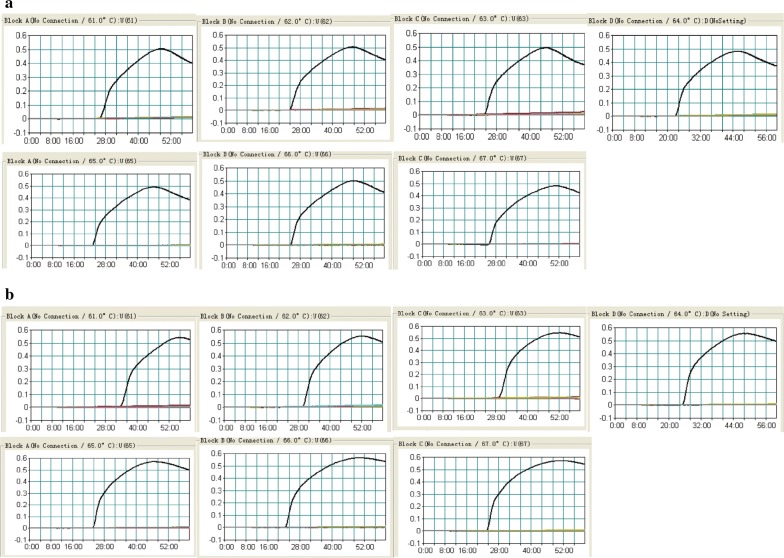
Optimal reaction temperature for *M. abscessus*- and *M. massiliense*-primer sets. The standard LAMP reactions for detection of *M. abscessus* (**a**) and *M. massiliense* (**b**) were monitored by real-time measurement of turbidity and the concentration of *M. abscessus*- and *M. massiliense*-DNA was 100 pg

To select the optimal amplification temperature for MABC–LAMP, the singlex *M. abscessus*- and *M. massiliense*-LAMP reactions were carried out from 61 to 67 °C with 1 °C intervals. The strains of *M. abscessus* (ATCC 19977) and *M. massiliense* (isolated-*massiliense*-001) were selected as the positive control at the level of 100 pg per reaction. Finally, 65 °C was selected as the best reaction temperature (Fig. 4). At this condition, no false amplifications occurred in standard *M. abscessus*-LAMP or *M. massiliense*-LAMP reaction.

### Feasibility of MABC–LAMP–LFD

**Fig. 5:**
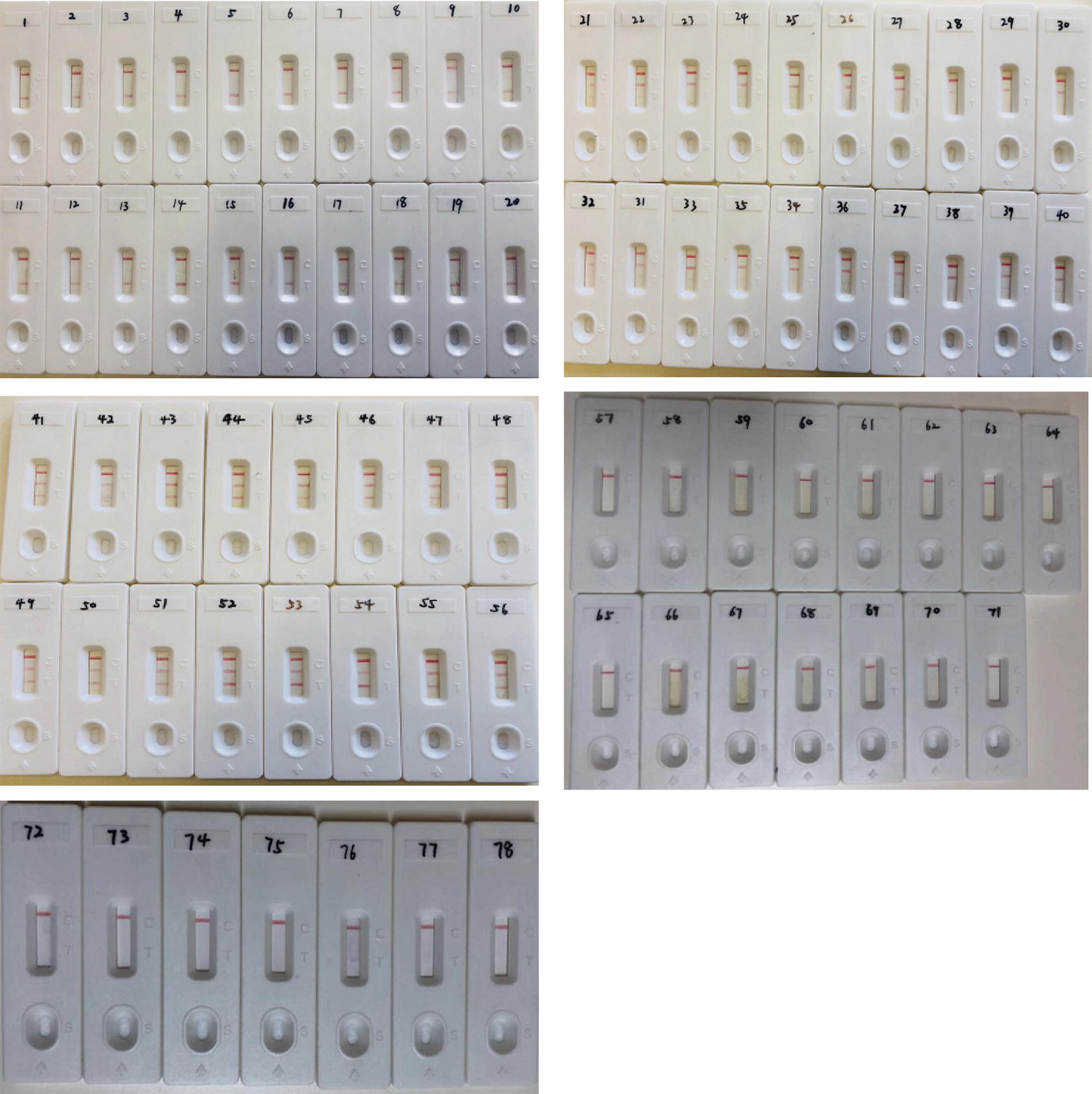
Feasibility of MABC–LAMP–LFD. 1–20, *M. abscessus*-DNA; 21–40, *M. massiliense*-DNA; 41–56, *M. abscessus* and *M. massiliense* mixed DNA; 57–71, non-*M. abscessus* complex isolates DNA; 72–78, non-mycobacterium isolates

**Fig. 6:**
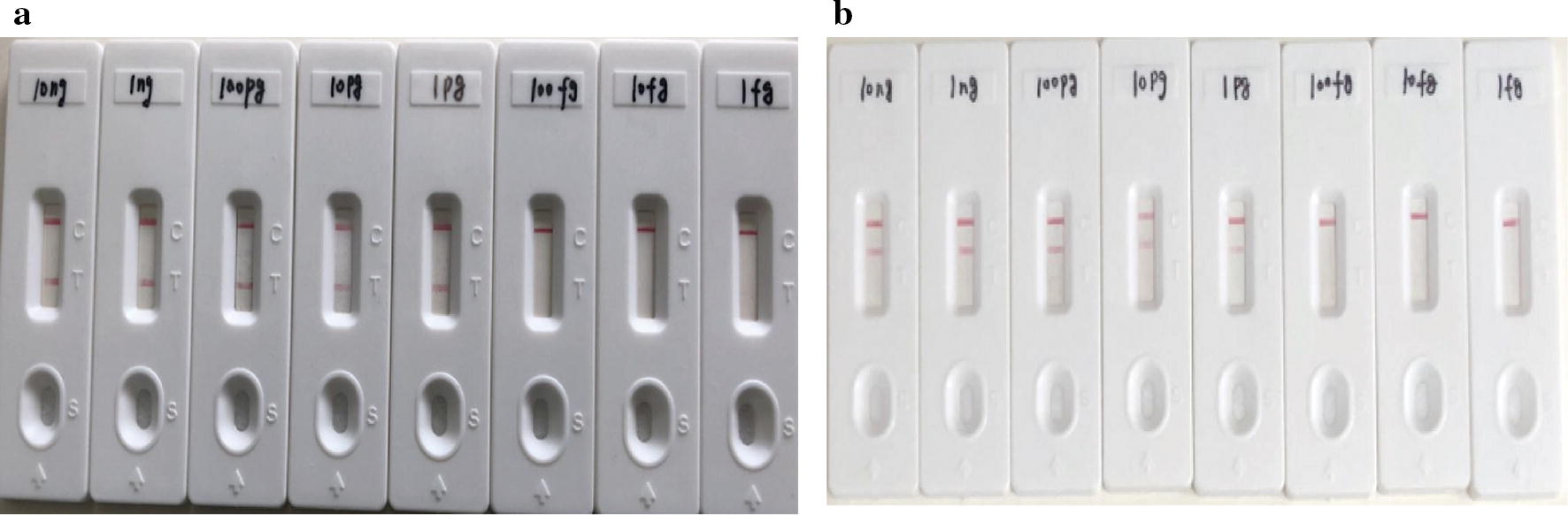
The limit of detection of MABC–LAMP–LFD assay. Serial dilutions of *M. abscessus*- and *M. massiliense*-DNA template (10 ng, 1 ng, 100 pg, 10 pg, 1 pg, 100 fg, 10 fg, 1 fg) were used** a** The LoD of* M. abscessus*** b** The LoD of* M. massiliense*

In MABC–LAMP–LFD positive reactions, clearly visible red lines for both test line I (TL I, for detection of *M. abscessus*) and test line II (TL II, for detection of *M. massiliense*) were observed. For negative reactions and blank controls, only the control lines were visible. Indeed, the testing of 20 *M. abscessus*, 20 *M. massiliense*, and 16 mixed clinical samples showed perfect concordance with the known species in each sample (Fig. 5). Additionally, all non-*M. abscessus* complex isolates and non-mycobacterial isolates tested did not bind either TL I or TL II, but only the control line (Fig. 5). Furthermore, serial dilutions of *M. abscessus* (ATCC 19977)- and *M. massiliense* (isolated strain)-genomic DNA were used to determine the limit of detection, which was shown to be as low as 1 pg DNA template for initiation of the MABC–LAMP reaction (Fig. 6).

## Discussion

*Mycobacterium abscessus* and *M. massiliense* are two important pathogens in the *M. abscessus* complex that cause human infections. The discriminatory detection of these species are associated with different drug-resistance and cure rate (Lee et al. 2015; Zelazny et al. 2009). The conventional methods for species identification based on phenotypic features cannot accurately delineate these two species, due to the close relationship between *M. abscessus* and *M. massiliense*, which makes differentiation of *M. abscessus* and *M. massiliense* challenging in most clinical microbiology laboratories. Recently, targeted gene (*hsp65*, *rpoB*, *ITS* and *secA1*) sequencing combined with phylogenomic analysis, *erm*(41) PCR-based assays, or multi-locus sequence analysis targeting 8 housekeeping genes have all been proposed as methods to identify them accurately (Macheras et al. 2011; Sassi et al. 2013). However, those methods require bacterial culture, PCR amplification, and/or gene sequencing, all of which are relatively time-consuming, costly, and may not be feasible in all clinical microbiology laboratories. In the current study, we described a simple, robust, accurate, rapid and cost-effective LAMP-based method to detect and distinguish *M. abscessus* and *M. massiliense* from clinical specimens directly. This method has several advantages over previously mentioned ones in terms of low equipment requirement (merely a heat block or water bath) and visualized results on a lateral flow strip, which increase the feasibility in resource-limited settings.

The MABC–LAMP–LFD method was able to rapidly and accurately identify *M. abscessus* and *M. massiliense* clinical isolates, as well as robustly detect mixed samples of the two strains. In addition, no false-positive detections occurred to other mycobacterial strains and non-mycobacterium isolates, therefore, this study represents a proof of concept for the use of the MABC–LAMP–LFD assay as a molecular diagnostic tool for detecting *M. abscessus* and *M. massiliense* with high sensitivity and specificity.

Differentiating *M. massiliense* from *M. abscessus* is clinically important, because they have different drug susceptibility profiles and treatment outcomes (Jeong et al. 2017). The cure rate of *M. massiliense* with clinical therapy is much higher in comparison with *M. abscessus infection*. In this study, the drug susceptibility testing against six antimicrobial agents for *M. abscessus* and *M. massiliense* isolates was performed respectively. *M. abscessus* showed high resistance to clarithromycin at day 3 and day 14 of incubation. Some *M. abscessus* strains have displayed intrinsic resistance to clarithromycin due to a point mutation at position A2058 or A2059 in the *rrl* gene region, while poor treatment outcomes of other *M. abscessus* infections have been attributed to inducible resistance via a functional erythromycin ribosomal methylase gene, *erm*(41). While the full-length *erm*(41) gene (522 bp) frequently confers inducible macrolide resistance in *M. abscessus*, the *M. massiliense erm*(41) gene contains several mutations, including a large C-terminal deletion that renders it nonfunctional. On basis of the difference between *M. abscessus erm*(41) gene and *M. massiliense erm*(41), we designed *M. abscessus*-specific LAMP primers and *M. massiliense*-specific LAMP primers respectively.

In conclusion, the LAMP-based method using two sets of primers combined with a label-based lateral flow biosensor shows rapid and accurate detection of *M. abscessus* and *M. massiliense* from clinical specimens and isolates. The incorporation of this method into the workflow of clinical laboratories will lead to decreased reliance on expensive and technologically demanding gene sequencing for identification of *M. abscessus* and *M. massiliense* isolates.

## Additional file

**Additional file 1.** MICs of 6 drugs for* M. abscessus* and* M. massiliense*.

## Abbreviations

*M. abscessus*: *Mycobacterium abscessus*
*M. massiliense*: *Mycobacterium massiliense*
RGM: rapidly growing mycobacteria
LAMP: loop-mediated isothermal amplification
LAMP–LFD: LAMP assay combined with a lateral flow dipstick
DST: drug susceptibility testing
USA: Sigma-Aldrich
LoD: limit of detection
